# Simultaneous stoma reinforcement and perineal reconstruction with biological mesh - A multicentre prospective observational study

**DOI:** 10.1016/j.amsu.2018.12.006

**Published:** 2018-12-21

**Authors:** Muhammad Imran Aslam, Naseer Baloch, Christopher Mann, Per J. Nilsson, Pierre Maina, Sanjay Chaudhri, Baljit Singh

**Affiliations:** a Department of Colorectal Surgery, Leicester General Hospital, University Hospitals of Leicester NHS Trust, Leicestershire, UK; b Center for Digestive Diseases, Karolinska University Hospital and Karolinska Institutet, Stockholm, Sweden; c Department of Surgery, Slagelse Hospital, Slagelse, Denmark

**Keywords:** Biological mesh, elAPE, Rectal cancer, Abdominoperineal excision, Strattice, Perineal hernia, Dynamic MRI

## Abstract

**Introduction:**

The optimal method for perineal reconstruction after extralevator abdominoperineal excision (elAPE) for low rectal cancer remains controversial. This study aimed to assess whether simultaneous perineal reconstruction and parastomal reinforcement with Strattice™ Reconstructive Tissue Matrix after elAPE could prevent hernia formation.

**Methods:**

In this prospective, multicentre, observational, non-comparative study of consecutive patients undergoing elAPE for low rectal cancer underwent simultaneous perineal reconstruction and colostomy site reinforcement with Strattice™ mesh. All patients underwent long course chemoradiotherapy prior to surgery and had excision of the coccyx. Patients were assessed for perineal wound healing at 7 day, 1, 3, 6 and 12 months, perineal and parastomal hernia defects on clinical and radiological assessment at 1 year following surgery.

**Results:**

19 patients (median age = 67 years, median BMI = 26, M:F = 11:8) were entered the study. 10 (52.6%) patients underwent laparoscopic elAPE. The median length of post-operative stay was 9 days. Complete wound healing was observed for 8(42%) patients at 1 month, 12(63%) at 3 months, and 19(100%) patients at 12 months. Median time for radiological and clinical assessment for hernias was 12 months. No perineal hernia was detected in 17 patients following CT assessment. Dynamic MRI was undertaken in 11 patients at 12 months and all showed no evidence of perineal hernia. 3 (16%) patients had a parastomal hernia detected radiologically. No mesh was removed during the 12 months follow up period.

**Conclusion:**

Perineal and parastomal reconstruction with biological mesh is a feasible approach for parastomal and perineal hernia prevention after laparoscopic and open elAPE.

## Highlights

• In this case series, consecutive patients underwent simultaneous perineal reconstruction and colostomy site reinforcement with Strattice™ biological mesh.
• Simultaneous perineal and parastomal reconstruction with Strattice™ mesh is an effective method of hernia prevention after elAPE.
• High quality prospective RCTs and national/international collaborative audits are required to compare this technique with others for perineal reconstruction.

## 1 Introduction

Although Miles first reported on the abdominoperineal excision (APE) more than 100 years ago a significant increase in interest in this operation has re-emerged since the publication of Holm et al. 2007 [1,2]. Today, the extra-levator abdominoperineal excision (elAPE) is a standard approach for a low rectal cancer [3]. The anticipated advantages of elAPE for reduced risk of involved lateral margins and inadvertent intraoperative bowel perforation leading to improved oncological outcomes [4], are counterbalanced by the increased risk of perineal wound complications [5]. The spectrum of perineal complication includes short-term problems such as wound infection and delayed healing and long-term complications such as perineal herniation or perineal sinus formation. The removal of the levator muscles, completely or in part, appears to increase the risk of negative perineal outcomes and this has led to the development of different techniques for perineal reconstruction following elAPE. One of these reported techniques involves the use of biological mesh for perineal reconstruction. Several case series, using different types of mesh and various end-points have been reported but there is limited data available regarding this technique [[6], [7]]. A systematic review did not show evidence for the use of biologic mesh in perineal reconstruction after proctectomy [8].

The elAPE inevitably leaves the patient with an end-colostomy. Cumulative incidence rates of parastomal hernia formation ranges up to 50% and a parastomal hernia may cause significant discomfort for the patient [9]. Although several techniques for parastomal hernia repair exist it may be stated that there is no “standard approach” and results with respect to recurrence rate are suboptimal. Thus, prevention of herniation is likely the best approach and promising results using a prophylactic mesh for trephine reinforcement have been reported [10,11]. A meta-analysis found 7 RCTs and other observational studies with a great deal of heterogeneity concluded that mesh prophylaxis at the time of stoma formation appears safe and effective in preventing parastomal hernia [12]. Strattice™ is a biological mesh derived from porcine dermis. The product is designed to provide biological scaffolding and promote strong tissue in-growth. The product has been reported to produce favourable results in abdominal wall reconstruction also in a contaminated surgical field [13].

The combination of both issues of perineal and parastomal hernia prevention into one study addresses a potentially common clinical scenario in elAPE patients. To address both these clinically relevant issues – perineal reconstruction with a biological mesh and stoma reinforcement in patients undergoing elAPE a prospective, multicentre, single arm, observational study using Strattice™ was undertaken. This study aimed to assess whether simultaneous perineal reconstruction and parastomal reinforcement with Strattice™ Reconstructive Tissue Matrix (Lifecell, Bridgewater NJ, USA) after elAPE could prevent hernia formation.

## 2 Methods

### 2.1 Study design

In this prospective, multicentre, observational study of non-comparative case series, consecutive patients undergoing elAPE for low rectal cancer underwent simultaneous perineal reconstruction and colostomy site reinforcement with Strattice™ biological mesh. Ethical permission for the study was obtained from local ethical committees for each centre. (University Hospitals of Leicester NHS Trust in UK, Centre for Digestive Diseases in Karolinska, Sweden and Department of Surgery Slagelse, Denmark). Lifecell provided Strattice™ mesh and funding for the administrative support required for the study. Study was registered at U.S. National Library of Medicine (Registration Number NCT01670851) and protocol was published at www.ClinicalTrials.gov and accessible at https://clinicaltrials.gov/ct2/show/NCT01670851.

This study was retrospectively registered on 26/06/2018 with UIN Research Registry (UIN researchregistry4217) with a title of “Simultaneous stoma reinforcement and perineal reconstruction with biological mesh - A multicentre prospective study”.

Outcome measures included perineal wound healing, perineal and parastomal hernia formation. Patients were assessed for perineal wound healing at time of discharge from hospital, day 7, 1 month, 3 months, 6 months and 12 months after surgery. Clinical assessments for perineal and parastomal hernia were made on standing and supine position on day 3, 6 months and 12 months. Radiological assessment for perineal and parastomal hernia was made at 12 months with Computed tomography (CT). Patient recruitment was commenced in July 2013 and finished in August 2014 for all three centres to allow 12 months of prospective recruitment. All patients were followed up for 12 months and follow up imaging was done as per study protocol.

### 2.2 Inclusion criteria

Patients undergoing curative resection for their primary cancer, able to attend all scheduled study visits, with life expectancy >2 years and who underwent neoadjuvant radiotherapy with or without chemotherapy were invited to participate in the study.

### 2.3 Exclusion criteria

Patients undergoing pelvic exenteration/extended resection, who received pelvic or perineal radiotherapy for another cancer, had sensitivity to porcine derived products or polysorbate sensitivity, participated in another trial or oncology trial involving biological therapy, had history of collagen disorders (System lupus erthematosis, rheumatoid arthritis, polypyositis, scleroderma, ankylosing spondylitis, Ehlers-Danlos or Marfan syndrome), system infections (HIV, Hepatitis C), renal failure, Child- Pugh B/C liver failure, chronic renal failure requiring haemodialysis or peritoneal dialysis, peritonitis or sepsis at the time of elAPE operation were excluded from participation.

### 2.4 elAPE operation

Patients underwent elAPE by using standardised technique. Surgery was performed by qualified colorectal specialist surgeons. After traditional mobilization of the left colon with vascular and bowel division, the rectum and mesorectum was dissected in the extramesorectal fascial plane. The abdominal dissection was stopped posteriorly at the sacrococcygeal junction, laterally just below the autonomic nerves and anteriorly just below the seminal vesicles in men or the cervix uteri in women. The abdominal part of the operation was completed first with either a laparoscopic or open approach and Strattice™ Reconstructive Tissue Matrix mesh (Lifecell, Bridgewater, New Jersey, USA) was inserted in sub-rectus pre-peritoneal space around the stoma site.

The perineal part of operation was completed for the most part in the prone position. The perineal dissection was commenced by making an elliptical incision around the anus and extended cranially to the coccyx. The anus was closed to reduce spillage with a purse string suture before the skin incision. The lateral dissection was continued in the subcutaneous fat, just outside the subcutaneous portion of the external anal sphincter. Following this plane, the levator ani muscle was identified on both sides and the dissection was continued along the outer surface of the levator ani muscles proximally until the insertion of the levator on to the pelvic sidewall. The lateral dissection was continued dorsally until the position of the coccyx was clearly palpated and the dissection proceeded on to the coccyx. The coccyx was disarticulated from the sacrum and the pelvis was entered by dividing Waldeyer's fascia. Then levators were divided laterally on both sides, from posterior to anterior, until the mesorectum becomes visible at the dorsal and lateral sides. A ‘pull through technique’ was preferred to retrieve the specimen. The pelvis was washed and drained via a tube drain. The pelvic floor was reconstructed with Strattice™ biological mesh, secured with interrupted monofilamentous, synthetic, non-absorbable suture (Polypropylene 2/0 sutures) and skin was closed with interrupted absorbable undyed 3/0 vicryl. The ischiorectal fat was approximated with interrupted absorbable sutures and the skin was closed. No antibiotics wash/sponge was used. No vacuum dressing or pressure dressings were applied. Non suction abdominal tube drain was placed in pelvis and no perineal drain was used. Above described operative technique was used by all recruiting centres.

### 2.5 Data collection

Data was collected prospectively. During the post-operative inpatient stay perineal wound and stoma were reviewed daily until discharge from hospital and data was recorded. The Southampton wound score [14] was used to assess perineal wound healing. Stomas and wounds were assessed by operating surgical team in hospital at day 7 and at the time of discharge from hospital. Stomas and wounds were assessed by surgical team in the outpatient settings at 1 month, 3 months, 6 months and 12 months. Clinical assessments for perineal and parastomal hernia were made on standing and supine position at 3, 6 and 12 months. Radiological assessment for perineal and parastomal hernia was made at 12 months with Computed tomography (CT).

Where feasible pelvic floor reconstruction was assessed using dynamic MRI for an abnormal pelvic floor descent and herniation. A perineal descent of more than 1 cm, or the presence of a hernial defect, was considered suboptimal. Dynamic MRI was performed 12 months after the elAPE procedure. The cine MRI sequence was a T2-weighted TrueFISP (True fast imaging with steady state precession) of a single, 10 mm slice, repeated at sub 0.5 s intervals to give a ‘CINE’ loop, and was acquired when the patient was asked to strain, thus demonstrating an active Valsalva manoeuvre [15]. T2-weighted sagittal and coronal views were obtained at rest in the supine position with the patient performing a Valsalva manoeuvre followed by imaging at rest, when the patient was not performing a Valsalva manoeuvre [16,17].

Data was collected and analysed in Excel worksheets This study work has been reported in line with the STROCSS criteria [18].

## 3 Results

Nineteen patients (median age = 67 years, median BMI = 26, M: F = 11:8) were entered the study from three centres (see Table 1). Ten (52.6%) patients underwent laparoscopic elAPE, eight (42%) had open surgery due to comorbidities (n = 6) and previous pelvic surgery (n = 2). One patient had laparoscopic converted to open surgery to facilitate TME dissection. There was no selection preference involved for laparoscopic vs. open surgery within institutions. Median post-operative stay was 9 days (range; 4–39 days). Patients who had their abdominal part of operation completed laparoscopically the median length of stay was 7.5 days (range; 4–39) whereas if the abdominal part of the operation was completed open the median length of stay was 11 days (range; 9–21). R0 resection was achieved for ypT1 (n = 5), ypT2 (n = 5), ypT3 (n = 7) and ypT4 (n = 2) rectal cancers. Table 2, Table 3 shows the demographics for patients undergoing elAPE in this study.

**Table 1:** Southampton wound score.

Wound	Grade	Grade	Appearance
Normal Wound healing	Grade 0	Grade 0	Normal healing
	Grade 1: Normal wound healing with mild bruising or erythema	Grade I-A	Some bruising
		Grade I-B	Considerable bruising
		Grade I-C	Mild erythema
Minor Wound Complication	Grade II: Erythema plus other signs of inflammation	Grade II-A	At one point
		Grade II-B	Around sutures
		Grade II-C	Along wound
		Grade II-D	Around wound
	Grade III: Clear or haemoserous discharge	Grade III-A	At one point only (<2 cm)
		Grade III-B	Along wound (>2 cm)
		Grade III-C	Large volume
		Grade III-D	Prolonged (>3 days)
Wound Infection	Grade IV: Pus	Grade IV -A	At one point only (<2 cm)
		Grade IV-B	Along wound (>2 cm)
Major Wound Complication	Grade V: Deep/severe wound infection	Grade V	Wound infection with or without tissue breakdown, haematoma, requiring aspiration

**Table 2:** Demographics for patients included in the study.

Criterion	Patient Characteristics	
Age	Median (Range) years	67 (50 - 84)
Gender	M:F	11:8
BMI	Median (Range)	26 (20–36)
ASA	ASA- I	01 (5.3%)
	ASA- II	11 (57.9%)
	ASA- III	07 (36.8%)
	ASA- IV	00
Smoking Status	Current smoker	00
	Ex -smoker	06 (31.6%)
	Non- smoker	13 (68.4%)
Comorbidities	Dibetes	04 (21.0%)
	COPD	04 (21.0%)
	Hypertension	07 (36.8%)
	Ischaemic heart disease	03 (15.7%)
	Cardiac arrthymia	02 (10.5%)
	Chronic kidney disease	03 (15.7%)
	Liver disorder	01 (5.3%)
	Peripheral vascular disease	01 (5.3%)
	Non GI cancer in the past	03 (15.7%)
Mode of Surgery	Open	7 (36.8%)
	Laparoscopic	10 (52.5%)
	Lap converted to open	02 (10.5%)
Assessment at 12 months	Clinical	19 (100%)
	CT scan	17 (89.5%)
	Dynamic MRI	11 (57.9%)

**Table 3:** Histology of resection and R0.

Criterion	Characteristics	Number (Percentage)
Tumour histology	ypT1	n = 5, 26.3%
	ypT2	n = 5, 26.3%
	ypT3	n = 7, 36.8%
	ypT4	n = 2, 10.5%
	ypN0	n = 12, 63.2%
	ypN1	n = 7, 36.8%
Curative Resection	R0	n = 19, 100%
Neoadjuvant Treatment	Chemoradiotherapy	n = 19, 100%

Complete perineal wound healing was observed for 8 (42%) patients at 1 month, 12 (63%) at 3 months, 16 (84%) at 6 months, and 19 (100%) patients at 12 months. One patient had pre-sacral abscess requiring antibiotics. One patient had a significant wound infection requiring a vacuum dressing. This patient had significant peripheral vascular disease and underwent vascular surgery for acute leg ischaemia 4 weeks after his rectal surgery. No mesh was removed during the 12 months follow up period. No mortality was reported at 12 months and none of the patients had grade III/IV Clavien-Dindo postoperative morbidity.

All patients had a clinical assessment for parastomal and perineal hernias at 3, 6 and 12 months. No perineal hernia was observed on clinical assessment at 3 and 6 and 12 months. Three patients had parastomal hernia on clinical examination and these were confirmed on CT scans. These hernias were clinically detectable only at 12 months follow up. 17 patients had CT scan of their abdomen and pelvis to assess for parastomal/perineal hernia at a median time of 12 months (range; 5–14 months). 2 patients could not have CT scan in the 12 months’ post-operative period either due to patient choice or contrast allergy. No perineal hernia was detected on CT scan for 17 patients.

**Fig. 1:**
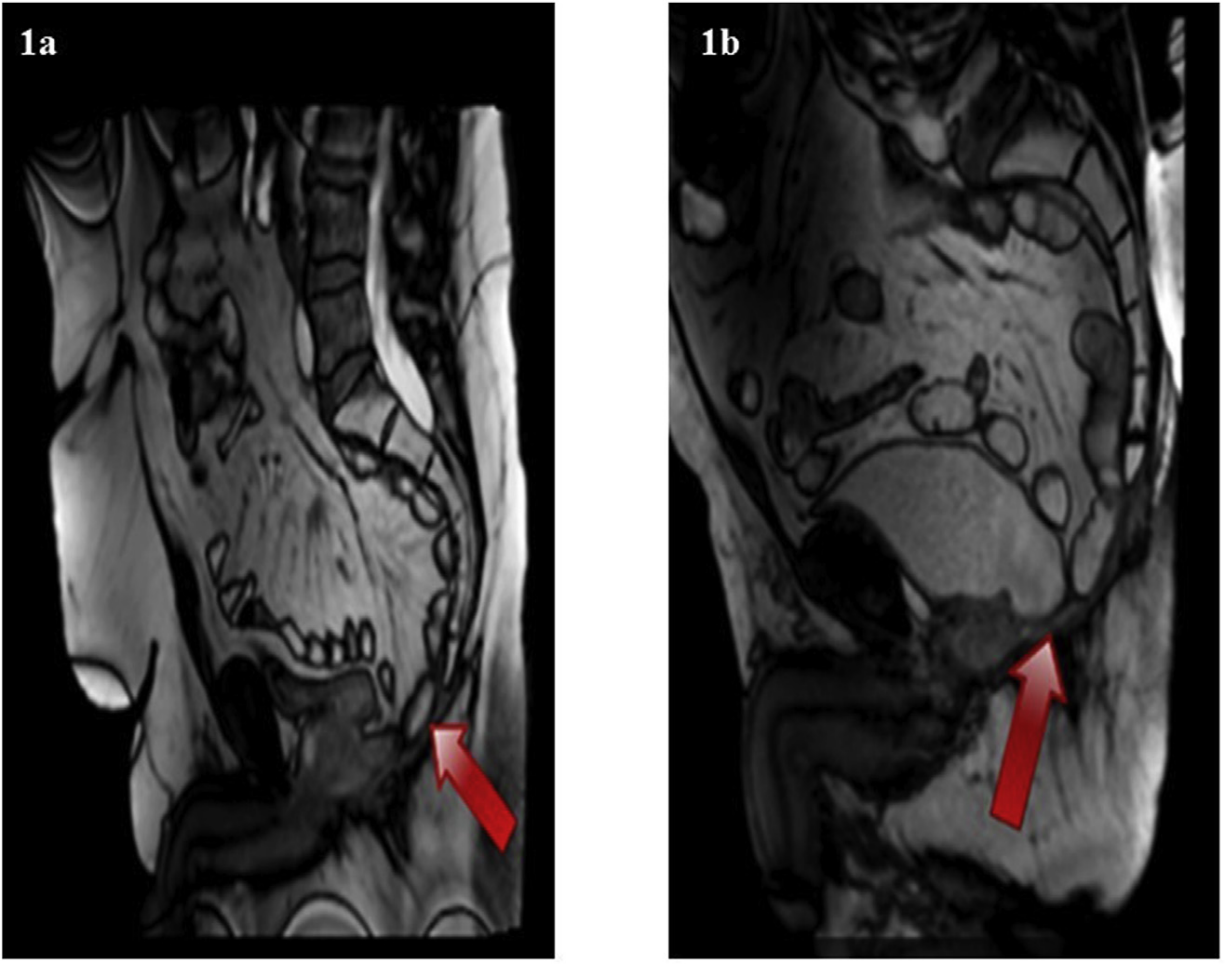
a and b illustrate static image capture from the dynamic MRI videos to illustrate the reconstructed pelvic floor after elAPE. Red arrows indicates reconstructed pelvic floor plate.

Dynamic MRI was undertaken for 11 patients at 12 months and showed no perineal hernia. One centre did not have the facility for Dynamic MRI imaging for their 5 patients. For other three patients who did not undergo Dynamic MRI imaging one patient was unfit, one was claustrophobic and one patient refused to undergo MRI imaging. Fig. 1a and b illustrate static image capture from the dynamic MRI videos to illustrate the reconstructed pelvic floor after elAPE. Red arrows indicates reconstructed pelvic floor plate.

3 (16%) patients had a parastomal hernia detected radiologically and these were confirmed clinically. The median size of the parastomal hernial defects was 45 × 61 mm. The hernia defect size on radiological assessments are given in Table 4. No patient required surgery to correct these hernias during 12 months follow up period, as they were asymptomatic. Two patients developed parastomal hernia after laparoscopic surgery and one patient developed hernia after open surgery.

**Table 4:** Patients undergoing radiological imaging for parastomal and perineal hernia with median time for CT scan, presence of parastomal hernia and size of hernia defect.

Patient No	Dynamic MRI (perineal) at one year	CT (Parastomal and Pelvis) months	Parastomal hernia (radiological)	Defect size (mm)
1	Y	12	N	0
2	Y	12	N	0
3	Y	12	N	0
4	N	12	N	0
5	N	12	N	0
6	N	12	Y	39 × 35
7	N	12	N	0
8	N	12	N	0
9	N-pacemaker	13	N	0
10	Y	12	N	0
11	Y	–	CT not done	N/A
12	Y	13	N	0
13	N-patient choice	7	Y	55 × 76
14	Y	14	N	0
15	Y	12	Y	45 × 61
16	Y	12	N	0
17	Y	–	CT not done	N/A
18	Y	13	N	0
19	N-patient unfit	5	N	0

## 4 Discussion

This study has shown that simultaneous prophylactic reinforcement of colostomy and perineum by using biological mesh during elAPE is feasible with complete wound healing in two third of patients at 3 months, in all patients at 12 months and no perineal hernia at 12 months. Given that elAPE produces a larger defect in the pelvic floor, perineal wound problems are reported in up to 57% of patients undergoing standard APE [19]. A systemic review [20] has reported no significant difference in perineal hernia rates or perineal wound infections between biological mesh and primary closure without mesh. Authors of this review had concluded that the overall volume and quality of evidence available regarding biologic mesh use for perineal reconstruction following elAPE was poor, with observational retrospective studies predominating [20]. There have been some attempts at comparative studies, but these too have been of low quality with a high risk of bias and confounding factors. The BIOPEX study however has shown that perineal reconstruction with a biological mesh led to a significantly lower incidence of perineal hernias at one year follow-up when compared to primary closure [21].

The biological mesh is usually placed as an inlay or bridge across the defect in the pelvic floor in close relation to the bony structures and sutured in 1-cm intervals to the origin of the levator muscles laterally [22]. The mechanism by which the use of a bridging prosthesis reduces perineal wound problems is not clear. The proposed mechanisms involve tissue regeneration, neovascularization, repopulation with fibroblasts, and therefore provides a scaffold for tissue incorporation [23,24]. It has been suggested that biological mesh allows native cellular ingrowth and promotes tissue remodelling, which in turn reduces perineal wound problems [23,25]. The biologic mesh may act as a physical barrier, supporting the pelvic contents (omentum, small bowel, and uterus) and minimizing the pressure on the skin and ischiorectal fat as they heal. Alternative to biological meshes, myocutaneous flaps, such as those derived from gluteus maximus [[26], [27], [28]] rectus abdominis, and latissimus dorsi muscles [26,29], have been used but are associated with donor-site morbidity, flap necrosis, prolonged operative time, additional resources, and increased cost. Head-to-head randomized trials or high-quality prospective cohort studies comparing biological with synthetic mesh, types of biologic mesh, and biologic mesh with myofasciocutaneous flaps are lacking, partly because there is no consensus among surgeons as to the optimal biologic mesh or optimal tissue flap. Furthermore, studies evaluating quality-of-life scores using validated tools demonstrated a favourable comparison to the reference population of patients with colorectal cancer who had undergone a standard APE, whereas patients who had undergone flap reconstruction had a lower quality of life score [30].

Present study showed low rates of parastomal hernia (16%) for patients undergoing prophylactic retro-rectus (sublay) biological mesh reinforcement. These results are similar to a previously published randomized control trial where the incidence of parastomal herniation was 12.5% for sublay biological mesh group [31]. Biomesh study group reviewed six systematic reviews, two randomized controlled trials (RCT), two case-controlled studies, and one technical report for the use of biological mesh for prophylactic reinforcement of stoma. Though, group reported a significant risk reduction of parastomal hernia development by biological mesh reinforcement of the permanent stoma at the index operation [32], it recommended further evaluations for choice of mesh material, mesh design and anatomical location. A large well-designed randomized clinical trial for parastomal reinforcement would be more likely to provide efficacy results of prophylactic mesh retro rectus (underlay) vs standard of care. Increased operative morbidity and technical difficulty (especially in laparoscopic cases) may exist with the prophylactic use of retro rectus mesh placement at the time of stoma creation and these would also need to be evaluated as secondary outcomes. This would likely require its own power analysis and sample number of patients which may ultimately differ from the number of patients required for the perineal reconstruction question.

The limitation of this study is the absence of a control arm to compare biological mesh reconstruction with other recognised techniques of perineal reconstruction. The fact that surgeons have different preferences for perineal reconstruction after extralevator proctectomy is not a reason for inability to carry out a randomized clinical trial which might help to better answer the question being examined in the present study. Without rigorous controlled randomized trials with long term follow up, the actual effectiveness of the various techniques of pelvic floor reconstruction remains controversial. In the case of the laparoscopic elAPE group biologic mesh vs. biologic mesh with gracilis muscle or vs. primary closure without mesh or flap would be feasible and helpful to answer the question of superiority of technique with respect to wound healing and hernia recurrence. In open elAPE either rectus flaps or gracilis flaps with biologic mesh or primary closure without mesh or flap could also be compared for non-inferiority in a randomized clinical trial. The present study could serve as a basis for a power analysis to determine the number of patients required in each arm to find a difference if one exists. Furthermore, there needs to be a focus on standardised definitions and reporting of perineal healing rates, perineal hernia, and functional outcomes following elAPE [33]. Due to small sample size, subgroup analysis for ASA grade, comorbidities, tumour location, histological stage/grade, duration of surgery and intraoperative blood loss were not analysed and reported in this manuscript.

Another limitation of this study is the length of follow up being limited to 12 months. To assess the long-term outcomes of perineal and parastomal reinforcement one would ideally need a longer period, such as 5–10 years. However, a review from Musters and colleagues has shown that the median time for perineal hernias to occur is 12months following elAPE [34]. A long-term follow-up study for patients undergoing pelvic floor reconstruction with a biologic mesh following elAPE showed very low perineal hernia rates [35]. In present study, the use of dynamic MRI scan has shown no descent of the pelvic floor suggesting effective repair with biological mesh. Dinnewitzer and colleagues [36] have reported similar results in their study of fourteen patients undergoing biological mesh reconstruction after elAPE with median dynamic MRI imaging at 31 months (range 19–56). They report no focal mesh defect, no damage on the suture line, and no perineal hernia.

Whilst this study did not look at the financial costs it could be argued that the use of a biological mesh is cost effective in reducing perineal and parastomal hernia formation and their subsequent repair. Furthermore, use of a biological mesh as the time of surgery adds little additional time to the overall operative time. Although not assessed it is likely this will also lead to a better quality of life. Cost-effectiveness, operative time, and quality of life, none of which are measured in the study, are interesting topics for follow-up studies.

## 5 Conclusion

Perineal and parastomal reconstruction with biological mesh is a feasible approach for parastomal and perineal hernia prevention after laparoscopic and open elAPE.

### 5.1 Provenance and peer review

Not commissioned, externally peer reviewed.

## Conflicts of interest

Nil.

## Authors contribution

MI Aslam: Study design, acquisition, analysis, interpretation of data, drafting of manuscript, final approval of the version to be published; Agreement to be accountable for all aspects of the work.

N Baloch: Design of the study, acquisition, analysis, interpretation of data, drafting of intellectual content, final approval of the version to be published; Agreement to be accountable for all aspects of the work.

C Mann: Acquisition, analysis, or interpretation of data, revision of intellectual content, final approval of the version to be published; Agreement to be accountable for all aspects of the work.

PJ Nilsson: Conception/design of the study, interpretation of data, revision of intellectual content, final approval of the version to be published; Agreement to be accountable for all aspects of the work.

P Maina: Conception/design of the study, acquisition, analysis, or interpretation of data, revision of intellectual content, final approval of the version to be published; Agreement to be accountable for all aspects of the work.

S Chaudhri: Conception/design of the study, acquisition, interpretation of data, revision of intellectual content, final approval of the version to be published; Agreement to be accountable for all aspects of the work.

B Singh: Conception/design of the study, acquisition, interpretation of data, revision of intellectual content, final approval of the version to be published; Agreement to be accountable for all aspects of the work.

## Sources of funding

Lifecell provided Strattice™ mesh and funding for the administrative support required for the study.

## Ethical approval

### Full title of study

Perineal reconstruction following extralevator abdominoperineal excision of rectum and simultaneous stoma sublay reinforcement (PRESSUR).

### Research sponsor

University Hospitals Leicester.

### Name of REC

NRES Committee East Midlands - Nottingham 1.

### REC reference number

13/EM/0106.

### Protocol number

CC0344.

### IRAS project ID

108553.

## Registration unique identifying

Researchregistry4217.

## Guarantor

Muhammad Imran Aslam.

Baljit Singh.
